# Associations between tinnitus and body composition: a cross-sectional study

**DOI:** 10.1038/s41598-024-67574-w

**Published:** 2024-07-16

**Authors:** Sang-Yoon Han, Sang-Yeon Lee, Myung-Whan Suh, Jun Ho Lee, Moo Kyun Park

**Affiliations:** 1 Department of Otolaryngology-Head and Neck Surgery, College of Medicine, Hanyang University, Seoul, Republic of Korea; 2 Department of Otorhinolaryngology-Head and Neck Surgery, Seoul National University Hospital, 101 Daehak-ro, Jongno-gu, Seoul, Republic of Korea; 3 Sensory Organ Research Institute, Medical Research Center, Seoul National University, Seoul, Republic of Korea

**Keywords:** Obesity, Visceral fat, Sarcopenia, Tinnitus, Chronic tinnitus, Neurological manifestations, Weight management, Obesity, Diseases, Endocrine system and metabolic diseases

## Abstract

The relationship between tinnitus and body composition in specific regions has not been extensively investigated. This study aimed to identify associations between tinnitus and body composition. Individuals with data on physical and otological examination findings, and bioelectrical impedance analysis were included from the ninth Korea National Health and Nutritional Examination Survey. They were divided into a tinnitus group and a non-tinnitus group. Participants with tinnitus were further classified into acute or chronic tinnitus group. The tinnitus group showed significantly higher body fat percentages in each region (arms: *P* = 0.014; legs: *P* = 0.029; trunk: *P* = 0.008; whole body: *P* = 0.010) and waist circumference (*P* = 0.007) than the non-tinnitus group, and exhibited lower leg muscle percentage (*P* = 0.038), total body fluid percentage (*P* = 0.010), and intracellular fluid percentage (*P* = 0.009) than the non-tinnitus group in men. Furthermore, men with chronic tinnitus showed a significantly higher trunk fat percentage (*P* = 0.015) and waist circumference (*P* = 0.043), and lower intracellular fluid percentage (*P* = 0.042) than their counterparts without tinnitus. No significant differences in body composition were observed among the groups in the female population. In men, body composition may be associated with tinnitus.

## Introduction

Tinnitus is an auditory perception that can be bothersome to patients^1^. It is categorized into two types: subjective tinnitus, which only the affected individuals can perceive, and objective tinnitus, which can be detected by physicians or others^1^. While objective tinnitus often stems from mechanical issues of vascular or muscular origins, many subjective tinnitus cases are attributed to hearing loss^1^. Nevertheless, subjective tinnitus also occurs in individuals with normal hearing. In such cases, research has found correlations between tinnitus and various physical conditions, including pain, infection, and sleep quality, as well as mental health issues like anxiety and depression^1,2^. Furthermore, subjective tinnitus has a significant relationship with brain metabolism and structure^3^. Therefore, some diseases that cause changes in the brain network through chronic inflammatory responses or are associated with structural and functional changes in the brain might be connected to subjective tinnitus^4,5^.

Some authors have also identified an association between tinnitus and obesity^2^. Özbey-Yücel and Uçar reviewed several articles and suggested that this relationship may be due to an elevated inflammatory response in obese patients^2^. Michaelides et al. found that pulsatile tinnitus is also linked to obesity and that weight reduction can be an effective treatment for this condition^6^. Furthermore, McCormack et al. determined that both body mass index (BMI) and body fat percentage were significantly associated with tinnitus^7^. However, they did not evaluate the type of obesity or the distribution of fat in obese patients.

Obesity is categorized into subtypes based on the distribution of body fat, with each subtype associated with distinct health conditions and disease characteristics^8,9^. The gold standard for evaluating body fat distribution is an imaging workup such as computed tomography or magnetic resonance imaging^10^. However, due to considerations of time and cost, dual-energy X-ray absorptiometry is commonly recommended for evaluating body composition, and bioelectrical impedance analysis (BIA) has also demonstrated high precision in this regard^10,11^. In this study, we aimed to investigate the relationship between body composition, as assessed by BIA, and tinnitus, as well as the chronicity of tinnitus in individuals with age-normative hearing levels. We specifically excluded individuals with hearing loss, which is known to be associated with tinnitus, from this study^1^. We also considered other factors that are linked to both tinnitus and obesity in order to identify the specific body composition patterns that are associated with tinnitus.

## Materials and methods

### Data source

We used data from the ninth Korea National Health and Nutritional Examination Survey (KNHANES). Data were extracted on age; sex; household income (quintile); weight; height; BMI; waist circumference; the body fat, muscle, and fluid percentage of each region; hypertension; diabetes; Patient Health Questionnaire-9 (PHQ-9)^12^; Generalized Anxiety Disorder-7 (GAD-7)^13^; history of dizziness; tinnitus; air-conduction hearing thresholds at 0.5 kHz, 1 kHz, 2 kHz, 4 kHz, and 8 kHz; and tympanic membrane status evaluated by tympanometry. The three individuals who selected “I don't remember” regarding their history of dizziness were classified as having no such history. Body composition measurements, including body fat, muscle, and fluid percentages, were conducted using bioelectrical impedance analysis (BIA) with the Inbody 970 device (Inbody, Seoul, Korea). We calculated the regional percentages of body fat, muscle, and fluid relative to total body mass by dividing the mass of each component in a given region by the total body weight. For the arms and legs, we determined the percentages of each composition by averaging the values from both the right and left sides. The PHQ-9 is a valid tool for evaluating depressive mood^12^, while the GAD-7 is a developed survey used for evaluating anxiety^13^. Air-conduction pure tone audiometry was carried out in a double-walled soundproof booth using an AD629 audiometer (Interacoustics, Assens, Denmark). Tympanometry was performed with a Titan IMP440 screener (Interacoustics, Assens, Denmark). Audiological assessments were conducted only on individuals aged 40 and above in the 9th KNHANES, a decision likely influenced by the increased prevalence of hearing loss in this age group^14^. All participants in the ninth KNHANES gave informed consent for this survey, which was approved by the Institutional Review Board (IRB) before the survey was conducted (IRB No. 2018-01-03-4C-A). The 9th KNHANES was conducted according to the examination guidelines provided by the Korea Disease Control and Prevention Agency. Additionally, this study adhered to the STROBE guidelines.

### Data availability

The ninth KNHANES data, which was used in our study, can be accessed through “https://knhanes.kdca.go.kr/knhanes/sub03/sub03_02_05.do”.

### Definition of normal hearing considering the age-norm hearing level

Since elderly individuals typically have higher hearing thresholds than young adults, with mean hearing thresholds—calculated by averaging frequencies at 0.5 kHz, 1 kHz, 2 kHz, and 4 kHz—being approximately 30 dB in their 70 s and around 40 dB in those over 80 years of age^15^, we used a 40 dB mean hearing threshold as the cutoff for hearing loss in our inclusion criteria.

### Inclusion and exclusion criteria of study subjects

From the participants in the ninth KNHANES, we selected subjects according to the following inclusion and exclusion criteria.

#### Inclusion criteria

- Individuals with data on age; sex; household income (quintile); weight; height; BMI; waist circumference; the body fat, muscle, and fluid percentage of each region; hypertension; diabetes; PHQ-9; GAD-7; history of dizziness; tinnitus; air-conduction hearing thresholds at 0.5 kHz, 1 kHz, 2 kHz, 4 kHz, and 8 kHz; and tympanic membrane status evaluated by tympanometry

#### Exclusion criteria

- Individuals who had mean hearing thresholds more than 40 dB
- Individuals with type B and C results from tympanometry, which represent abnormal tympanic membranes, such as a tympanic membrane perforation, otitis media, or a retracted tympanic membrane.

### Definition of tinnitus and subject classification

In the ninth KNHANES, tinnitus was assessed through a survey and defined as present when a participant reported the symptom lasting for five minutes or more within the past year. Acute tinnitus was further defined as lasting for five minutes or more but less than 6 months, while chronic tinnitus was defined as having a duration of 6 months or more.

The study subjects were classified into two groups—the tinnitus group and the non-tinnitus group—based on the presence or absence of tinnitus. Those with tinnitus were also categorized into chronic and acute subgroups according to the chronicity of the condition.

### Definition of obesity and central obesity

Obesity was defined according to the guidelines provided by the World Health Organization, using total body fat percentage with a cutoff of 25% for males and 35% for females^16^. Central obesity was diagnosed based on the Korean Society for the Study of Obesity guidelines, using waist circumference with a threshold of 90 cm or over for males and 85 cm or over for females^17^.

### Statistical analysis

Analysis of variance and the chi-square test were conducted to compare the variables among the groups. Multinomial logistic regression analysis was performed to compare the categorical variables among the groups after adjusting other factors. Multivariate analysis of covariance was performed for significant variables identified in the univariable study to control for other covariates and adjusted mean value of body composition in each part of the body. A *P-*value < 0.05 was considered to indicate statistical significance. All statistical analyses were performed with SPSS version 25.0 (IBM Corp., Armonk, NY, USA). The symbols *, **, and *** were used for *P*-values less than 0.05, 0.01, and 0.001, respectively, in the figures.

## Results

### Demographic factors, economic status, underlying diseases, and audiological characteristics of each group

**Figure 1:**
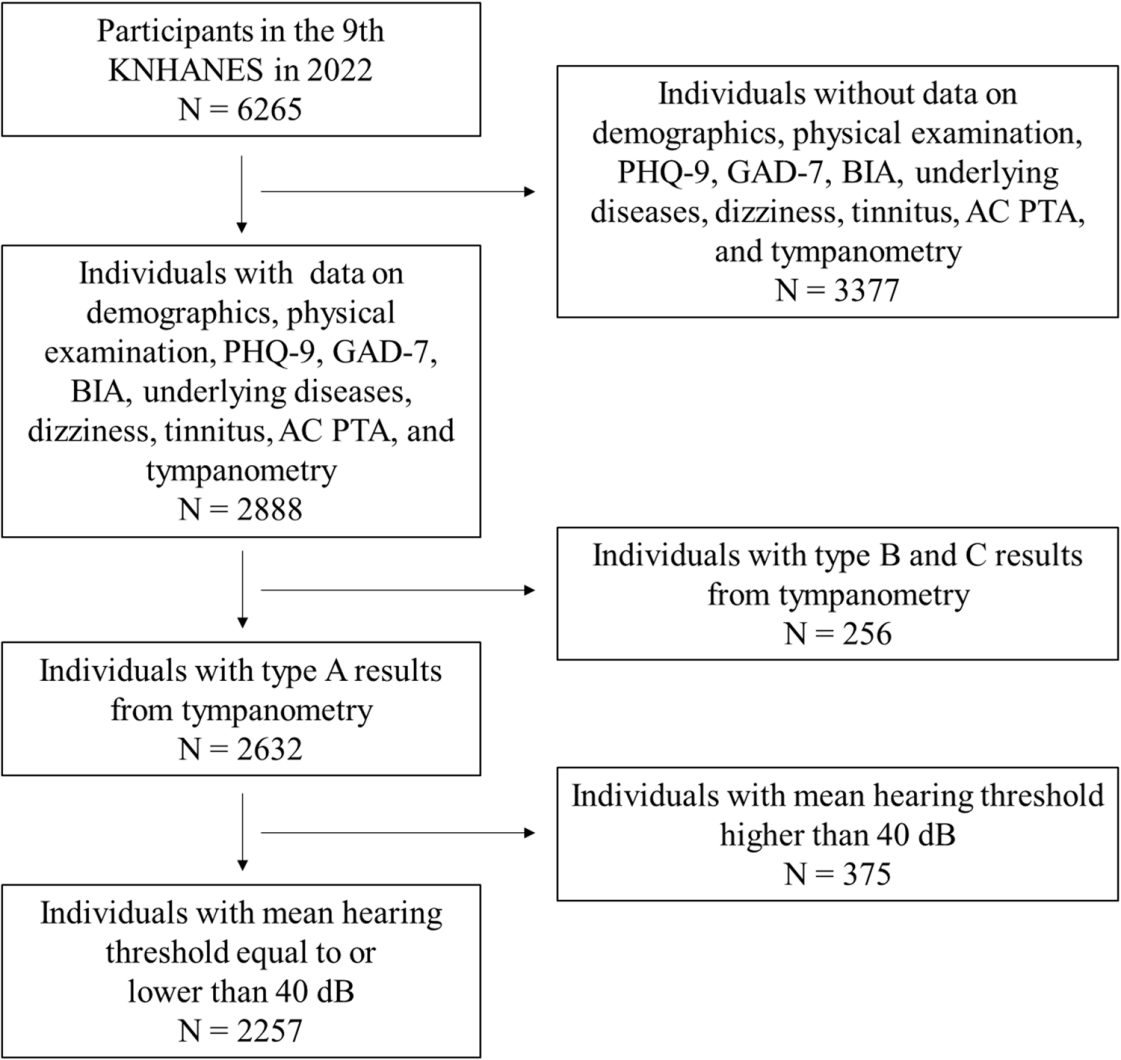
Inclusion process of subjects in this study from ninth Korea National Health Examination and Nutrition Survey. *KNHANES* Korea National Health and Nutrition Examination Survey, *N* number, *PHQ-9* Patient Health Questionnaire-9, *GAD-7* generalized anxiety disorder-7; *BIA* bioelectrical impedance analysis, *AC* air-conduction, *PTA* pure tone audiometry.

Among the 6265 KNHANES participants who were initially considered, 3377 were screened out due to lack of data. Subsequently, 256 individuals with abnormal tympanic membranes were excluded. To control for the effect of hearing loss, we excluded 375 individuals who had a hearing threshold higher than 40 dB in the better ear. Ultimately, 2257 individuals were included in this study (Fig. 1). Of these, 204 were classified into the tinnitus group, and 2125 were classified into the non-tinnitus group.

The mean age of the tinnitus group (60.22 ± 11.30 years) was older than that of the non-tinnitus group (57.09 ± 10.68 years, *P* < 0.001). Tinnitus prevalence was higher among men (11.76%) than among women (6.90%, *P* < 0.001). The mean household income was significantly lower in the tinnitus group (quintile mean = 2.91 ± 1.31) than in the non-tinnitus group (quintile mean = 3.34 ± 1.36, *P* < 0.001). Hypertension was more prevalent in the tinnitus group (36.68%) than in the non-tinnitus group (29.30%, *P* = 0.030). However, there was no significant difference in the prevalence of diabetes between the groups (*P* = 0.215). The tinnitus group showed higher PHQ-9 (3.00 ± 4.42) and GAD-7 scores (2.57 ± 4.02) than the non-tinnitus group (PHQ-9: 2.10 ± 3.28, *P* < 0.001; GAD-7: 1.92 ± 3.22, *P* < 0.001). The history of dizziness was higher in the tinnitus group (50.25%) than in the non-tinnitus group (33.33%, *P* < 0.001). The mean hearing level was worse in the tinnitus group (21.66 ± 9.44 dB) than in the non-tinnitus group (16.81 ± 8.52 dB, *P* < 0.031) (Table 1).

**Table 1:** Age, sex, household income, underlying disease, and mean hearing level in the tinnitus and non-tinnitus groups.

Total	Groups		*P*-value
	Tinnitus group (N = 199)	Non-tinnitus group (N = 2058)	
Age (years)	60.22 ± 11.30	57.09 ± 10.68	** < 0.001**
Sex (M:F)	105 : 94	788 : 1270	** < 0.001**
Household income (quintile)	2.91 ± 1.31	3.34 ± 1.36	** < 0.001**
Hypertension (%)	36.68%	29.30%	**0.030**
Diabetes mellitus (%)	15.08%	12.05%	0.215
PHQ-9 score	3.00 ± 4.42	2.57 ± 4.02	** < 0.001**
GAD-7 score	2.10 ± 3.28	1.92 ± 3.22	** < 0.001**
History of dizziness	50.25%	33.33%	** < 0.001**
Mean hearing level (dB)	21.76 ± 9.43	16.95 ± 8.53	**0.031**

*N* number, *M* male, *F* female, *PHQ-9* Patient Health Questionnaire-9, *GAD-7* generalized anxiety disorder-7.

Significant values (*P* < 0.05) are in bold.

After controlling for age, there was a significant difference only in mean hearing level (*P* < 0.001) between the groups in male population (Table 2). Household income (*P* = 0.108), hypertension (*P* = 0.080), and diabetes (*P* = 0.497), PHQ-9 score (*P* = 0.053), and GAD-7 score (*P* = 0.083) were not significantly different between the groups in the male population. And, household income *(P* = 0.002), PHQ-9 score *(P* = 0.002), GAD-7 score *(P* < 0.001), history of dizziness (*P* < 0.001), and mean hearing level (*P* = 0.004) were significantly different among the groups in the female population, while other variables such as hypertension and diabetes did not show significant differences (Table 2).

**Table 2:** Comparison of household income, underlying disease, and mean hearing level, after controlling for age, between the tinnitus and non-tinnitus groups in each sex.

Male	Groups		*P*-value
	Tinnitus group (N = 105)	Non-tinnitus group (N = 788)	
Household income (quintile)	3.23 ± 0.12	3.44 ± 0.04	0.108
Hypertension (%)	aOR = 1.465, 95% CI = 0.955–2.247		0.080
Diabetes mellitus (%)	aOR = 1.199, 95% CI = 0.710–2.025		0.497
PHQ-9 score	2.26 ± 0.31	1.68 ± 0.11	0.053
GAD-7 score	2.10 ± 0.28	1.53 ± 0.10	0.083
History of dizziness	aOR = 1.483, 95% CI = 0.966–2.275		0.072
Mean hearing level (dB)	22.22 ± 0.66	18.26 ± 0.24	** < 0.001**

Female	Groups		*P*-value
	Tinnitus group (N = 94)	Non-tinnitus group (N = 1270)	
Household income (quintile)	2.84 ± 0.13	3.25 ± 0.04	**0.002**
Hypertension (%)	aOR = 1.299, 95% CI = 0.772–2.188		0.325
Diabetes mellitus (%)	aOR = 0.828, 95% CI = 0.400–1.712		0.610
PHQ-9 score	4.02 ± 0.36	2.35 ± 0.10	** < 0.001**
GAD-7 score	3.34 ± 0.36	2.14 ± 0.10	**0.002**
History of dizziness	aOR = 3.079, 95% CI = 1.990–4.765		** < 0.001**
Mean hearing level (dB)	18.14 ± 0.68	16.13 ± 0.18	**0.004**

*N* number, *aOR* adjusted odds ratio, *PHQ-9* Patient Health Questionnaire-9, *GAD-7* generalized anxiety disorder-7, *CI* confidence interval.

Significant values (*P* < 0.05) are in bold.

### Comparison of body fat, muscle, and fluid percentage of each region between the groups.

Since the distribution and amount of fat, muscle, and fluid significantly differ according to sex^18^, we analyzed their sex-specific associations with tinnitus.

#### Men

Men with tinnitus exhibited a higher percentage of body fat in each region (*P* = 0.005 for the total body; *P* = 0.007 for the arms; *P* = 0.006 for the trunk; *P* = 0.011 for the legs), a higher waist circumference (*P* = 0.011), and less muscle mass in each region (*P* = 0.024 for the arms, *P* = 0.010 for the legs). Additionally, they had lower total body and intracellular fluid levels (*P* = 0.007 for total body fluid; *P* = 0.002 for total intracellular fluid) (Table 3).

**Table 3:** Comparison of the fat, muscle, and fluid percentages of each region between the tinnitus and non-tinnitus groups.

Male	Variables	Groups		*P*-value
		Tinnitus group (N = 105)	Non-tinnitus group (N = 788)	
	Total body fat (%)	26.25 ± 5.04	24.68 ± 5.39	**0.005**
	Arm fat (%)	1.64 ± 0.53	1.49 ± 0.55	**0.007**
	Trunk fat (%)	13.87 ± 3.10	12.96 ± 3.21	**0.006**
	Leg fat (%)	3.72 ± 0.56	3.55 ± 0.64	**0.011**
	Arm muscle (%)	4.21 ± 0.32	4.30 ± 0.37	**0.024**
	Leg muscle (%)	11.40 ± 1.09	11.69 ± 1.11	**0.010**
	Body fluid (%)	54.30 ± 3.75	55.42 ± 4.00	**0.007**
	Intracellular fluid (%)	33.55 ± 2.36	34.37 ± 2.51	**0.002**
	Extracellular fluid (%)	20.75 ± 1.50	21.06 ± 1.57	0.062
	Waist circumference (cm)	91.26 ± 9.54	88.92 ± 8.80	**0.011**
	BMI (kg/m^2^)	25.06 ± 3.48	24.81 ± 3.32	0.482

Female	Variables	Groups		*P*-value
		Tinnitus group (N = 94)	Non-tinnitus group (N = 1270)	
	Total body fat (%)	33.81 ± 5.79	33.29 ± 5.79	0.446
	Arm fat (%)	2.43 ± 0.64	2.37 ± 0.61	0.362
	Trunk fat (%)	17.05 ± 3.25	16.78 ± 3.37	0.446
	Leg fat (%)	5.00 ± 0.77	4.96 ± 0.72	0.606
	Arm muscle (%)	3.26 ± 0.33	3.30 ± 0.30	0.201
	Leg muscle (%)	9.72 ± 1.12	9.97 ± 1.19	0.050
	Body fluid (%)	48.63 ± 4.29	49.02 ± 4.25	0.391
	Intracellular fluid (%)	29.83 ± 2.65	30.13 ± 2.66	0.301
	Extracellular fluid (%)	18.80 ± 1.68	18.89 ± 1.64	0.585
	Waist circumference (cm)	81.76 ± 9.66	81.27 ± 9.38	0.625
	BMI (kg/m^2^)	23.72 ± 3.58	23.77 ± 3.46	0.897

*N* number, *BMI* body mass index.

Significant values (*P* < 0.05) are in bold.

#### Women

Female participants exhibited a marginal difference in leg muscle percentage between the groups (*P* = 0.050), but no other differences were observed in terms of fat, muscle, and fluid percentages in various body regions (Table 3).

### Multivariable analysis of fat and muscle distribution

**Figure 2:**
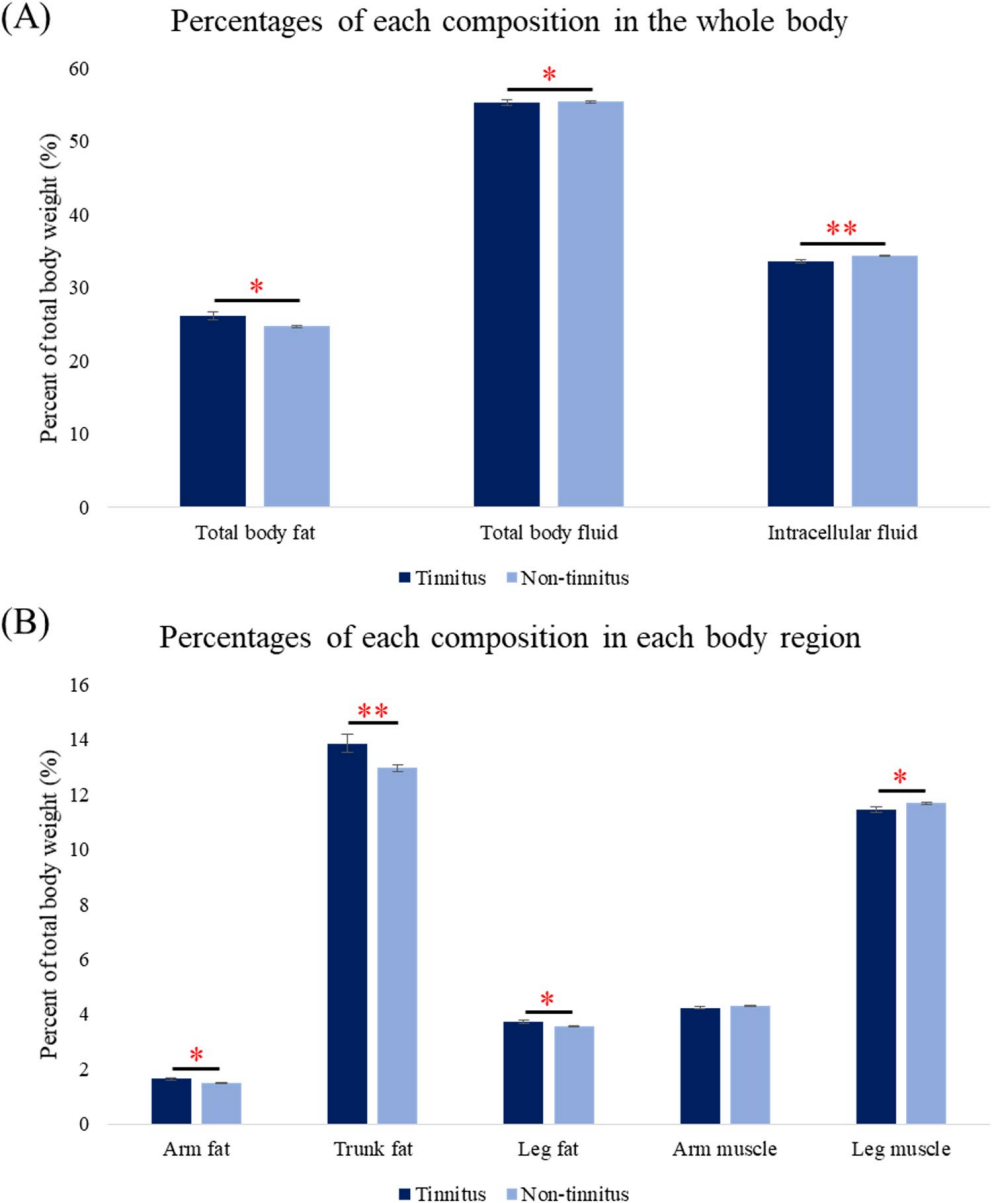
Comparison of age- and mean hearing level-adjusted fat and fluid percentages in the whole body (**A**) and each region (**B**) between the tinnitus and non-tinnitus groups in the male population. * Statistically significant difference between the tinnitus group and the non-tinnitus group; *P < 0.05; **P < 0.01.

We conducted a multivariable analysis using body composition variables that were significantly different in each region according to univariable analysis. Other variables that differed significantly between the groups were included as covariates (Table 2). After adjusting for age and mean hearing level, the tinnitus group exhibited higher total body fat percentage (*P* = 0.010), arm fat percentage (*P* = 0.014), leg fat percentage (*P* = 0.029), and trunk fat percentage (*P* = 0.008). In addition, the tinnitus group had lower leg muscle percentage (*P* = 0.038), body fluid percentage (*P* = 0.010), and intracellular fluid percentage (*P* = 0.009) compared to the non-tinnitus group in the male population (Fig. 2). Furthermore, waist circumference was significantly greater in the tinnitus group (mean waist circumference = 91.42 ± 0.88 cm; 95% CI 89.69–93.15 cm) than in the non-tinnitus group (mean waist circumference = 88.90 ± 0.32 cm; 95% CI 88.28–89.52 cm, *P* = 0.007).

The female population did not exhibit any differences in leg muscle percentage after adjusting for other factors (*P* = 0.552).

### Subgroup analysis for chronic tinnitus and acute tinnitus

We categorized the tinnitus cohort into two groups: those with chronic tinnitus and those with acute tinnitus, based on the duration of their symptoms.

#### Demographic factors

Among the tinnitus group, 152 were classified into the chronic tinnitus group and 47 were classified into the acute tinnitus group. The mean age of the chronic acute group (60.78 ± 11.01 years) was older than that of the other groups, followed by the acute tinnitus group (58.43 ± 12.12 years), and then the non-tinnitus group (57.09 ± 10.68 years, *P* < 0.001). The gender distribution of each group was different (chronic tinnitus M:F = 86:66; acute tinnitus M:F = 19:28; non-tinnitus M:F = 788:1270, *P* < 0.001).

#### Men

The chronic tinnitus, acute tinnitus, and non-tinnitus groups showed statistically significant differences only in hearing thresholds (*P* < 0.001) and exhibited no differences in household income, diabetes, hypertension, PHQ-9 score, GAD-7 score, and history of dizziness after controlling for age in the male population (Table 4).

**Table 4:** Comparison of household income, underlying disease, and mean hearing level, after controlling for age, among the chronic tinnitus, acute tinnitus, and non-tinnitus groups in each sex.

Male	Groups			*P*-value
	Chronic tinnitus group (N = 86)	Acute tinnitus group (N = 19)	Non-tinnitus group (N = 788)	
Household income (quintile)	3.27 ± 0.14	3.07 ± 0.29	3.44 ± 0.04	0.228
Hypertension (%)	aOR = 1.396 95% CI = 0.874–2.229	aOR = 1.821 95% CI = 0.71–4.675	–	0.193
Diabetes mellitus (%)	aOR = 1.194 95% CI = 0.674–2.117	aOR = 1.225 95% CI = 0.395–3.794	–	0.797
PHQ-9 score	2.34 ± 0.35	1.90 ± 0.73	1.68 ± 0.11	0.192
GAD-7 score	2.20 ± 0.31	1.57 ± 0.65	1.53 ± 0.10	0.102
History of dizziness	aOR = 1.509 95% CI = 0.947–2.404	aOR = 1.368 95% CI = 0.528–3.542	–	0.202
Mean hearing level (dB)	22.48 ± 0.73	21.05 ± 1.55	18.26 ± 0.24	** < 0.001**

Female	Groups			*P*-value
	Chronic tinnitus group (N = 66)	Acute tinnitus group (N = 28)	Non-tinnitus group (N = 1270)	
Household income (quintile)	2.86 ± 0.16	2.77 ± 0.24	3.25 ± 0.04	**0.008**
Hypertension (%)	aOR = 0.748 95% CI = 0.409–1.365	aOR = 0.812 95% CI = 0.301–2.196	–	0.587
Diabetes mellitus (%)	aOR = 0.842 95% CI = 0.370–1.919	aOR = 0.772 95% CI = 0.176–3.392	–	0.865
PHQ-9 score	3.85 ± 0.43	4.41 ± 0.66	2.35 ± 0.10	** < 0.001**
GAD-7 score	3.12 ± 0.43	3.85 ± 0.66	2.14 ± 0.10	**0.004**
History of dizziness	aOR = 2.526 95% CI = 1.524–4.188	aOR = 5.125 95% CI = 2.161–12.155	–	** < 0.001**
Mean hearing level (dB)	19.23 ± 0.81	15.59 ± 1.24	16.13 ± 0.18	**0.001**

*N* number, *PHQ-9* Patient Health Questionnaire-9, *GAD-7* generalized anxiety disorder-7, *aOR* adjusted odds ratio, *CI* confidence interval.

Significant values (*P* < 0.05) are in bold.

The body fat percentage in various regions was compared among the chronic tinnitus group, the acute tinnitus group, and the non-tinnitus group within the male population. Significant differences were observed in the total body fat percentage (*P* = 0.019), arm fat percentage (*P* = 0.026), leg fat percentage (*P* = 0.039) and trunk fat percentage (*P* = 0.024) among the groups (Table 5). Furthermore, there were significant differences in leg muscle percentage (*P* = 0.036), total body fluid (*P* = 0.025), and intracellular fluid percentage (*P* = 0.007) across the groups (Table 5). Additionally, significant differences were found in the mean waist circumference (*P* = 0.040) among these groups (Table 5).

**Table 5:** Comparison of the fat, muscle, and fluid percentages of each region among the chronic tinnitus, transient tinnitus, and non-tinnitus groups.

Male	Variables	Groups			*P*-value
		Chronic tinnitus group (N = 86)	Transient tinnitus group (N = 19)	Non-tinnitus group (N = 788)	
	Total body fat (%)	26.27 ± 5.00	26.18 ± 5.35	24.68 ± 5.39	**0.019**
	Arm fat (%)	1.65 ± 0.53	1.62 ± 0.52	1.49 ± 0.55	**0.026**
	Trunk fat (%)	13.88 ± 3.08	13.81 ± 3.28	12.96 ± 3.21	**0.024**
	Leg fat (%)	3.72 ± 0.55	3.72 ± 0.61	3.55 ± 0.64	**0.039**
	Arm muscle (%)	4.21 ± 0.32	4.23 ± 0.37	4.30 ± 0.37	0.077
	Leg muscle (%)	11.409 ± 1.10	11.39 ± 1.07	11.69 ± 1.11	**0.036**
	Body fluid (%)	54.29 ± 3.74	54.37 ± 3.89	55.42 ± 4.00	**0.025**
	Intracellular fluid (%)	33.54 ± 2.34	33.60 ± 2.24	34.37 ± 2.51	**0.007**
	Extracellular fluid (%)	20.75 ± 1.51	20.77 ± 1.46	21.06 ± 1.57	0.176
	Waist circumference (cm)	91.31 ± 9.69	91.06 ± 9.08	88.92 ± 8.80	**0.040**
	BMI (kg/m^2^)	25.06 ± 3.60	25.06 ± 2.97	24.81 ± 3.32	0.781

Female	Variables	Groups			*P*-value
		Chronic tinnitus group (N = 66)	Transient tinnitus group (N = 28)	Non-tinnitus group (N = 1270)	
	Total body fat (%)	34.04 ± 5.67	33.25 ± 6.13	33.29 ± 5.79	0.586
	Arm fat (%)	2.46 ± 0.64	2.37 ± 0.66	2.37 ± 0.61	0.537
	Trunk fat (%)	17.24 ± 3.23	16.61 ± 3.31	16.78 ± 3.37	0.530
	Leg fat (%)	5.00 ± 0.74	5.01 ± 0.86	4.96 ± 0.72	0.875
	Arm muscle (%)	3.25 ± 0.33	3.28 ± 0.35	3.30 ± 0.30	0.404
	Leg muscle (%)	9.60 ± 1.14	10.02 ± 1.03	9.97 ± 1.19	**0.043**
	Body fluid (%)	48.47 ± 4.20	49.01 ± 4.54	49.02 ± 4.25	0.589
	Intracellular fluid (%)	29.70 ± 2.60	30.15 ± 2.77	30.13 ± 2.66	0.440
	Extracellular fluid (%)	18.77 ± 1.64	18.86 ± 1.80	18.89 ± 1.64	0.837
	Waist circumference (cm)	81.89 ± 9.75	81.47 ± 9.61	81.27 ± 9.38	0.870
	BMI (kg/m^2^)	23.85 ± 3.46	23.42 ± 3.88	23.77 ± 3.46	0.847

*N* number, *BMI* body mass index.

Significant values (*P* < 0.05) are in bold.

**Figure 3:**
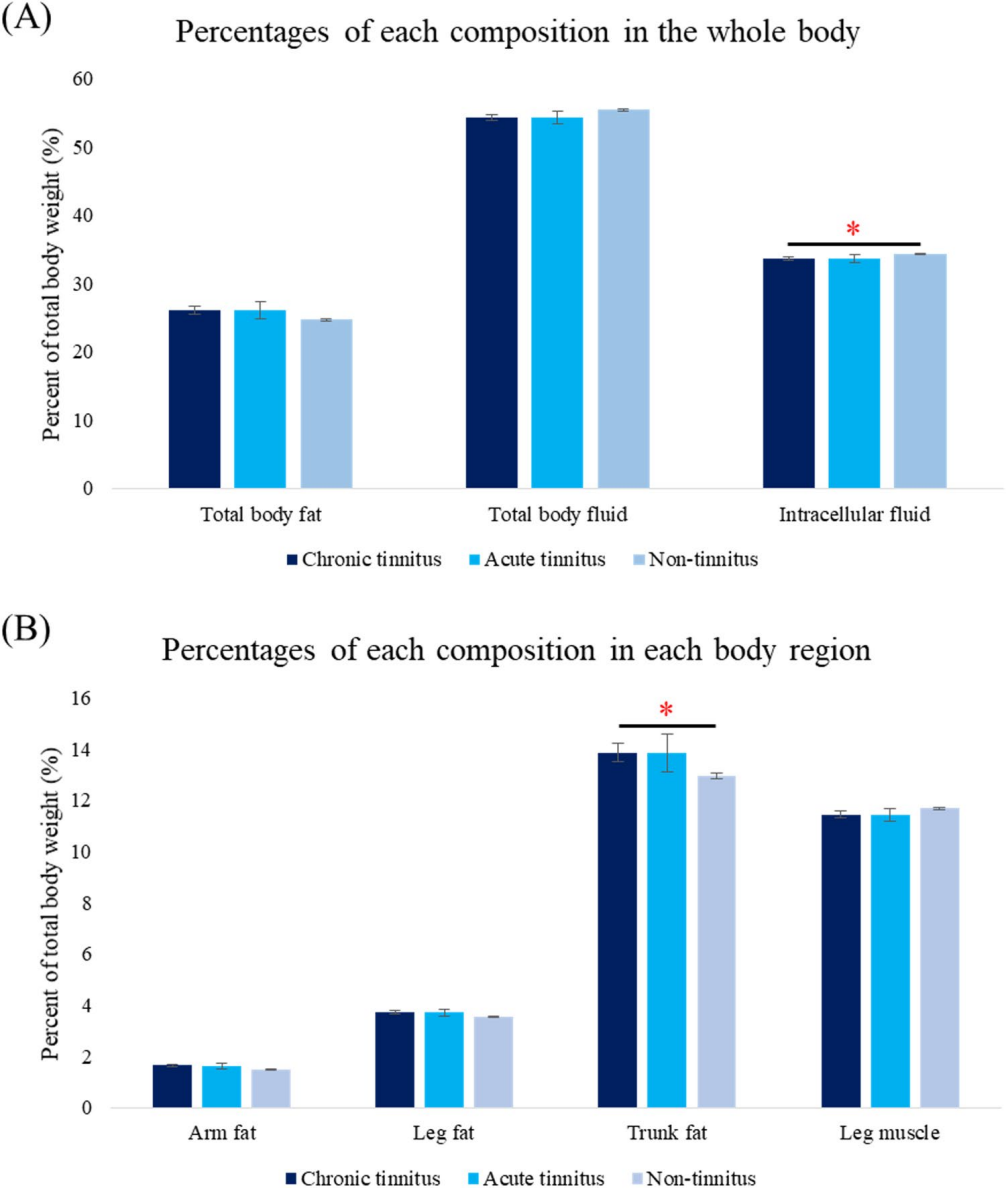
Comparison of age- and mean hearing level -adjusted fat and fluid percentages in the whole body (**A**) and each region (**B**) among the chronic tinnitus, acute tinnitus, and non-tinnitus groups in the male population. *Statistically meaningful difference between the chronic tinnitus group and the non-tinnitus group; *P < 0.05.

After controlling for age and mean hearing level, significant differences were observed among the groups in the male population for total body fat percentage (*P* = 0.035), arm fat percentage (*P* = 0.049), trunk fat percentage (*P* = 0.030), waist circumference (*P* = 0.028), body fluid percentage (*P* = 0.036), and intracellular fluid percentage (*P* = 0.032) (Fig. 3). In the post-hoc analysis, the chronic tinnitus group showed significantly higher trunk fat percentage (mean = 13.86 ± 0.35%; 95% CI 13.17–14.55%) and waist circumference (mean = 91.41 ± 0.97 cm; 95% CI 89.51–93.32 cm), and significantly lower intracellular fluid (mean = 33.64 ± 0.27 cm; 95% CI 33.10–34.18) than the non-tinnitus group (mean trunk fat percentage = 12.96 ± 0.11%; 95% CI 12.73–13.18%, *P* = 0.015; mean waist circumference = 88.90 ± 0.32 cm; 95% CI 88.28–89.52 cm, *P* = 0.043; mean intracellular fluid percentage = 34.35 ± 0.09%; 95% CI 34.18–34.53% , *P* = 0.042) in the male population.

#### Women

In the female population, significant differences were observed between the chronic and acute tinnitus groups in terms of household income (*P* = 0.008), PHQ-9 score (*P* < 0.001), GAD-7 score (*P* = 0.004) history of dizziness (*P* < 0.001), and mean hearing thresholds (*P* = 0.001) after adjusting for age (Table 4).

Leg muscle percentage (*P* = 0.043) was the only body composition-related variable that showed a significant difference between the chronic and acute tinnitus groups in the female population (Table 5). However, this relationship was no longer statistically significant after controlling for age, household income, PHQ-9 score, GAD-7 score, history of dizziness, and mean hearing threshold (*P* = 0.600).

### Prevalence and chronicity of tinnitus in obesity or central obesity

#### Prevalence of tinnitus

Since we demonstrated that body fat percentage and waist circumference were significantly associated with tinnitus, we classified obesity using a cut-off of 25% body fat for males and 35% body fat for females. Central obesity was defined with a cut-off of 90 cm waist circumference for males and 80 cm for females.

Among the subjects, 46.92% and 40.47% were classified as obese in the male and female populations, respectively. The prevalence of tinnitus was much higher in males with obesity (19.03%) than in those without obesity (8.72%, odds ratio (OR) = 2.18, *P* < 0.001). In females, there were no differences in tinnitus prevalence between obese and non-obese patients (*P* = 0.280).

Additionally, 46.25% of the male population and 33.50% of the female population had central obesity. Males with central obesity showed a higher prevalence of tinnitus (16.67%) compared to those without central obesity (10.60%, OR 1.57, *P* = 0.030). In contrast, females did not show different prevalence rates according to their obesity status (*P* = 0.212).

Binary logistic regression analysis was performed for obesity and central obesity, including age and mean hearing level for males. Tinnitus was significantly associated with both obesity (adjusted OR (aOR) 2.093, 95% CI 1.357–3.227, *P* = 0.001) and central obesity (aOR 1.707, 95% CI 1.115–2.613, *P* = 0.014).

#### Proportion of chronic or acute tinnitus

The same evaluation was performed for chronic and acute tinnitus. The chronic tinnitus, acute tinnitus, and non-tinnitus groups occupied 13.13%, 2.86%, and 84.00%, respectively, in males with obesity, which was different from the proportions in non-obese patients (chronic tinnitus: 6.54%; acute tinnitus: 1.48%; non-tinnitus: 91.98%, *P* = 0.001). Additionally, males with central obesity also showed significantly different proportions of chronic, acute, and non-tinnitus groups (chronic tinnitus: 12.35%; acute tinnitus: 1.94%; non-tinnitus: 85.71%) compared to those without central obesity (chronic tinnitus: 7.29%; acute tinnitus: 2.29%; non-tinnitus: 90.42%, *P* = 0.037). In contrast, female individuals with obesity or central obesity did not show a difference in the proportion of chronic tinnitus or acute tinnitus compared to those without obesity (*P* = 0.280) or central obesity (*P* = 0.215).

Multinomial logistic regression analysis was performed including age, mean hearing level, and obesity or central obesity in the male population. The chronic tinnitus group was significantly associated with obesity (aOR 1.927, 95% CI 1.207–3.077, *P* = 0.006) or central obesity (aOR 2.115, 95% CI 1.318–3.393, *P* = 0.002).

## Discussion

Previous studies have reported an association between obesity and tinnitus. However, the relationships between tinnitus and specific types of obesity or the amount of fat in each body region have not been thoroughly investigated. In our study, we demonstrated that body fat percentage, sarcopenia in the lower limbs, body fluid percentage, and intracellular water percentage are significantly associated with tinnitus, particularly in male individuals. Additionally, chronic tinnitus was found to be significantly associated with trunk fat percentage and waist circumference. It also significantly associated with obesity and central obesity.

Obesity is strongly associated with an increased risk of adult diseases, including cardiovascular disease, hypertension, and insulin resistance, as well as type 2 diabetes^19^. It also promotes systemic inflammation, which can lead to various types of systemic damage^19^. Furthermore, fat accumulated around the trunk is indicative of visceral fat, which is considered the most problematic site of fat accumulation^20,21^. Vogel et al. showed that abdominal fat is more closely associated with oxygen tension, which correlates with pro-inflammatory gene expression^21^. This reaction may increase cardiovascular risk in individuals with central obesity^8,9^. Since tinnitus is significantly related to systemic inflammation^5^, it is possible that tinnitus could be a side effect of upper body obesity. Moreover, considering the clinical importance of visceral fat, the chronicity of tinnitus may be more influenced by visceral obesity, as assessed by the percentage of trunk fat and waist circumference.

In addition, the metabolism and structure of brain regions associated with tinnitus are affected by obesity^22,23^. Previous studies have shown that patients with obesity have reduced total brain and gray matter volume, which is attributed to neuroinflammation. Furthermore, the fronto-temporal regions, which are involved in the noise cancellation pathway, are also associated with obesity^3,22,23^. Since tinnitus is linked to the noise cancellation pathway^3^, these structural changes in the brain may contribute to the onset and persistence of tinnitus.

A study revealed that obesity-related brain changes differ between sexes, with significant reductions in gray matter volume observed in the thalamus, caudate nucleus, putamen, globus pallidus, hippocampus, and nucleus accumbens exclusively in male patients with obesity^24^. These areas are closely associated with tinnitus and are involved in the noise-cancellation pathway^25,26^. Therefore, early detection of obesity-related tinnitus can be beneficial in preventing further brain changes that may contribute to the chronicity of tinnitus. Further research is needed to identify the specific brain regions associated with tinnitus in obese individuals and to understand the mechanisms underlying their relationship.

Our results also indicated a significant association between lower leg muscle mass and tinnitus. Additionally, multivariable analysis indicated that intracellular fluid levels tend to decrease as the duration of tinnitus increases. Given that total body fluid and intracellular fluid are indicative of body muscle mass^27^, the higher levels of these fluids in the non-tinnitus group suggest that sarcopenia may be a risk factor for tinnitus. Furthermore, the lower leg muscle mass might have the most significant impact on tinnitus, considering that skeletal muscle mass is greater in the lower body than in the upper body^28^. The link between tinnitus and sarcopenia could be attributed to functional changes in the brain. Suo et al. demonstrated that progressive resistance training is significantly associated with the functional connectivity of the posterior cingulate cortex and hippocampus, which are key regions of the default mode network (DMN)^29,30^. The DMN is involved in self-referential mental processes during the resting state^3,30,31^ and is part of the triple network, which has been shown to be significantly related to tinnitus^3,30^. Considering the relationship between sarcopenia and the DMN, resistance training aimed at preventing sarcopenia may also be helpful for preventing tinnitus.

Moreover, some studies have demonstrated that brain-derived neurotrophic factor (BDNF) is significantly associated with resistance training in men, but not in women^32,33^. Based on these findings, sarcopenia is significantly related to lower BDNF levels in men, which may lead to altered functional connectivity in the DMN in the male population, potentially inducing tinnitus. Additionally, compared to male sarcopenia, female sarcopenia can be accompanied by hormonal changes after menopause^34^. In this study, we could not evaluate menopause due to a lack of information. Although females performed muscle training, their skeletal muscle can decrease after menopause^34^, probably leading to different sarcopenia-associated features in females. Further research is necessary to elucidate the precise relationship between sarcopenia and tinnitus, as well as the associated brain changes.

In this study, there was no difference in BMI among the groups when considering tinnitus and its duration. This finding diverges from those reported in other previous studies. It is important to note that BMI is associated not only with fat content, particularly visceral fat, but also with muscle mass^35^. Therefore, an elevated BMI may result from increased levels of either fat or muscle. Given that our results indicate a link between tinnitus and both central obesity and sarcopenia, BMI fails to accurately reflect an individual’s body composition in the presence of these conditions. Therefore, measures that more specifically assess visceral fat, such as trunk fat percentage and waist circumference, should be considered as potential risk factors for tinnitus rather than BMI.

BIA is commonly used to evaluate body composition^10^. However, it tends to yield different results for various ethnic groups^36^. Therefore, distinct equations should be applied for different ethnicities. Some recent studies have reported that the body composition of Asians and Koreans can be accurately evaluated using a recently developed equation and device^10,37^, one of which was applied in this study. Consequently, clinicians should consider these factors before assessing body composition and providing interventions.

This study revealed that tinnitus was associated with age and mean hearing level in both male and female populations, and was related to household income, depressive mood, anxiety, and dizziness only in the female group. Hearing loss is one of the major causes of tinnitus. Age-related degenerative changes in the brain and age-related hearing loss are also major causes of tinnitus^1,4^. Some previous studies have identified associations between tinnitus and factors such as patient income, physical health, and mental health^1,38,39^. These findings are consistent with our study, which reveals an association of tinnitus with age, household income, depressive mood, anxiety, and dizziness. Additionally, some previous studies about the association between psychological symptoms and tinnitus in each gender showed that females with tinnitus have more psychological and physical comorbidities and tinnitus in females was significantly associated with depressive mood or anxiety, while in males it was not^40–42^. Based on these previous studies and our results, tinnitus in females is more associated with psychological and physical symptoms, while in males, it is less associated with those symptoms.

The main limitation of our study was its cross-sectional design, which relied on a database from a previously conducted survey. Because the database lacked additional information, such as the characteristics and types of tinnitus, we were unable to determine the specific features of tinnitus. Given that subjective tinnitus is more common than objective tinnitus, there may be a relationship between upper-body obesity and subjective tinnitus. To clarify the characteristics of tinnitus associated with upper-body obesity, further studies with more comprehensive tinnitus information are required.

Another limitation of our study is the inclusion of both the acute tinnitus group and the chronic tinnitus group for evaluating tinnitus-related body composition. Since we could not evaluate the exact duration of tinnitus and whether the tinnitus is ongoing in each patient, and the number of participants in the acute tinnitus group was small, it was difficult to demonstrate the factors associated with tinnitus in the acute stage. Therefore, we conducted the analysis of tinnitus-related body composition by including both the acute and chronic tinnitus groups because about 80% of the acute tinnitus can progress to chronic tinnitus^43,44^, and some causes and risk factors are shared between both chronic and acute tinnitus^1^. However, since the neural networks and auditory pathways of patients with acute tinnitus are significantly different from those of patients with chronic tinnitus^45^, further studies with a larger number of acute tinnitus cases may be helpful in evaluating the factors associated with body composition in the acute tinnitus stage.

This study also had the limitation of including patients with mild hearing loss classified by WHO classification^46^. Since hearing level was significantly associated with tinnitus, we tried to control the hearing level. However, when using a 25 dB cutoff for defining hearing loss, the number of participants significantly decreased due to a higher proportion of hearing loss in older individuals. Therefore, we used a 40 dB cutoff for defining hearing loss and adjusted the hearing level for the analysis of the association between tinnitus and body composition.

Additionally, we were unable to establish causality between upper limb obesity and tinnitus due to the limitations inherent in cross-sectional studies. A prospective cohort study would be beneficial in clarifying the causality between these conditions.

## Conclusion

Body composition was significantly associated with tinnitus in the male population, and central obesity was linked to chronic tinnitus in males. Furthermore, assessing the risk of central obesity in males with chronic tinnitus could be advantageous for early detection of central obesity, thereby mitigating additional cardiovascular risk.

## Data Availability

The data used in our study can be accessed through “https://knhanes.kdca.go.kr/knhanes/sub03/sub03_02_05.do”.
